# The Role of Reactive Oxygen Species in the Life Cycle of the Mitochondrion

**DOI:** 10.3390/ijms21062173

**Published:** 2020-03-21

**Authors:** Paola Venditti, Sergio Di Meo

**Affiliations:** Dipartimento di Biologia, Università di Napoli Federico II, Complesso Universitario Monte Sant’Angelo, Via Cinthia, I-80126 Napoli, Italy; sergio.dimeo@unina.it

**Keywords:** mitochondrial turnover, ROS, PGC-1, Nrf2, mitochondrial fission, mitochondrial fusion, transition pore, mitophagy

## Abstract

Currently, it is known that, in living systems, free radicals and other reactive oxygen and nitrogen species play a double role, because they can cause oxidative damage and tissue dysfunction and serve as molecular signals activating stress responses that are beneficial to the organism. It is also known that mitochondria, because of their capacity to produce free radicals, play a major role in tissue oxidative damage and dysfunction and provide protection against excessive tissue dysfunction through several mechanisms, including the stimulation of permeability transition pore opening. This process leads to mitoptosis and mitophagy, two sequential processes that are a universal route of elimination of dysfunctional mitochondria and is essential to protect cells from the harm due to mitochondrial disordered metabolism. To date, there is significant evidence not only that the above processes are induced by enhanced reactive oxygen species (ROS) production, but also that such production is involved in the other phases of the mitochondrial life cycle. Accumulating evidence also suggests that these effects are mediated through the regulation of the expression and the activity of proteins that are engaged in processes such as genesis, fission, fusion, and removal of mitochondria. This review provides an account of the developments of the knowledge on the dynamics of the mitochondrial population, examining the mechanisms governing their genesis, life, and death, and elucidating the role played by free radicals in such processes.

## 1. Introduction

Chemical species possessing one or more unpaired electrons, called free radicals, are continuously produced in living organisms as by-products of normal metabolic processes that are essential to the cell.

Free radicals and nonradical species, resulting from a partial reduction of a poorly reactive biradical such as oxygen and referred to as reactive oxygen species (ROS), are recognized to play a crucial role in the regulation of critical physiological processes and be involved in the development of pathological conditions [1].

ROS include species, such as the hydroxyl radical (^•^OH), which can cause irreversible damage to macromolecules with consequent loss of their function, and species, such as superoxide (O_2_^•‒^) and hydrogen peroxide (H_2_O_2_) that are less reactive, which can act as signaling molecules regulating a wide variety of physiological functions [2].

Like ROS, nitrogen-containing species, called reactive nitrogen species (RNS), play a dual role since they can be either harmful or beneficial to living systems. They include nitric oxide (NO^•^), which is relatively unreactive and is known as a regulator of important physiological processes [3], and peroxynitrite (ONOO^−^), a powerful oxidant, which can mediate cellular toxicity [4]. ONOO^−^ cytotoxicity depends not only on its direct action but also on its evolution to secondary free radical intermediates such as nitrogen dioxide and carbonate radicals [4].

The extent of the production of free radical affects the role they play in living systems. If produced to a large extent, they cause oxidative damage and tissue dysfunction while, if produced in moderate quantities, they act as molecular signals that induce adaptive responses useful for the organism. It is worth noting that the terms “large” and “moderate”, lacking effective quantitative redox biology techniques, only indicates relative differences in radical production.

Initially, free radicals were believed to have only harmful effects and be implicated in various diseases, but subsequently, it was recognized that they could also play regulatory roles [5].

In the cell, ROS are generated in several systems localized on the plasma membrane, in the cytosol, in the peroxisomes, and on plasma, mitochondrial, and endoplasmic reticulum membranes [1]. Although it was recently argued that mitochondria are not a major source of ROS in the cell [6], the fact that mitochondrial oxygen consumption is from 90% to 95% of cellular consumption and that in vitro measurements during basal respiration indicated that about 1–2% from that pool can be converted to O_2_^•−^ [7], has provided a strong argument in favor of mitochondria as the main source of this oxygen radical.

Due to their high capacity for ROS production mitochondria can also be detrimental to the cell. Mitochondrial ROS could damage cellular components, such as lipids, proteins, and DNA making them dysfunctional and dangerous for the life of the cell and causing degenerative processes underlying diseases and aging [1]. Mitochondria are the major targets of ROS detrimental effects, representing the trigger for cellular dysfunctions. The damage inflicted by free radicals to mitochondrial components decreases their ability to perform their functions lowering the homeostatic ability of tissues to adapt to the destabilizing effects of external and internal stresses.

However, several results indicate that paradoxically ROS-activated mechanisms are operative to provide cellular protection against excessive dysfunction, in which mitochondrial systems seem to play a major role. For example, the mitochondria are provided with the highest capacity to produce free radicals and, therefore, the more damaged can be eliminated and replaced by newly formed mitochondria [8]. It is also likely that the cell needs feedback from mitochondria to coordinate mitochondrial biogenesis and/or removal during the division cycle. The idea that the same radicals produced by mitochondria or other cellular sources play a crucial role in the mitochondrial genesis and removal is now widely shared.

In this review, after briefly examining some aspects of the relationship between mitochondria and ROS, we will give an account of the developments of the knowledge on the dynamics of the mitochondrial population, examining the mechanisms governing genesis, life, and death of mitochondria and elucidating the complex role played by free radicals in such processes

## 2. ROS–Mitochondria Interaction

Mitochondria are double-membrane-bound subcellular organelles that are found in most of the eukaryotic organisms and are crucial for the life of the cell. Most mammalian cells contain several hundred to more than a thousand mitochondria whose size, shape, and abundance vary dramatically in different cell types. Their principal function is to supply most of the energy required for the various endergonic reactions carried out by the cell, in the form of adenosine-5′-triphosphate (ATP) synthesized through electron transport and oxidative phosphorylation.

The mitochondrial content of a given tissue largely reflects its specific demands for respiratory energy. Thus, cells that have a greater demand for ATP such as hepatic, muscular, gastric, and nerve cells contain about 1–2 thousand mitochondria, whereas blood cells and skin cells have very few or no mitochondria [9]. On the other hand, because cellular function relies on adequate ATP supply, under different energy demands linked to changes in physiological and environmental conditions, the content of mitochondria can change to modify the ATP production and maintain cellular function.

The downside is that the flow of electrons along the mitochondrial respiratory chain leading to ATP production is also primarily responsible for the mitochondrial production of ROS.

A mitochondrial H_2_O_2_ production was first observed in 1966 by Jensen, who found that antimycin-insensitive oxidation of nicotinamide adenine dinucleotide (NADH) and succinate by bovine heart submitochondrial particle was coupled with H_2_O_2_ production [10]. Soon after, it was demonstrated that under aerobic condition H_2_O_2_ was generated by pigeon heart mitochondria in the presence of succinate [11]. The discovery that mitochondria contain their own superoxide dismutase (SOD) [12], an enzyme able to convert O_2_^•−^ to H_2_O_2_, suggested that H_2_O_2_ generated within mitochondria arose from the dismutation of O_2_^•−^. The subsequent detection of the mitochondrial generation of superoxide radical anion [13] confirmed that O_2_^•−^ produced by mitochondria is a stoichiometric precursor of H_2_O_2_

Although O_2_^•−^ generation is a continuous and physiological occurrence, the maintenance of metabolic functions is possible because mitochondria have a highly efficient antioxidant defense system able to scavenge a great part of the ROS produced either within or diffusing into mitochondria from other cellular sites [14]. This means that the systems evolved to protect mitochondria against endogenously produced ROS can also scavenge ROS produced by other cellular sources. Therefore, the mitochondria may serve as an intracellular sink for ROS, which contrasts with the role usually recognized for the organelles as ROS producers. The modulation of intracellular ROS levels by the mitochondrial antioxidant system is crucial for cellular homeostasis as different ROS levels can induce different biological responses. In normal conditions, such a system contributes with extra-mitochondrial systems to maintain cellular ROS at physiological levels for cell signaling, sustaining cellular proliferation and differentiation and activating stress-responsive survival pathways [15]. Conversely, when ROS production by mitochondrial and extramitochondrial sources overwhelms the capacity of the mitochondrial antioxidant systems, components of the respiratory chain, enzymes of the Krebs cycle, and other proteins can be inactivated, leading to mitochondrial dysfunction and cell death [14].

The effects of ROS-induced damage of mitochondrial DNA (mtDNA) are also particularly relevant. Due to less efficient repair mechanisms, mtDNA is more susceptible than nuclear DNA to damage from ROS. Moreover, the sites of ROS production and mtDNA location, which is principally attached to the matrix side of the inner mitochondrial membrane, overlap, and so mtDNA must be repaired under ROS “bombardment”, affecting DNA repair proteins and lowering the efficacy of DNA repair [16]. Such a situation promotes the formation of mtDNA-protein crosslinks mediated by ROS, which is one of, if not the most serious, form of DNA damage [17]. The maintenance of mtDNA can be crucial to the proper functioning of mitochondria so that mtDNA damage can play an important role in the pathogenesis of mitochondria- or ROS-related diseases.

## 3. Mitochondrial Life Cycle

To maintain cells throughout their life, mitochondria undergo various dynamic changes, allowing them to maintain their functionality and provide energy, and avoid harm to the host cells. Mitochondrial dynamics disturbances lead to neuronal malfunctioning or degeneration. In the cell, mitochondria pursue their own semi-autonomous “life-cycle” consisting of several steps. For every one of these processes, a wealth of mechanistic information is available from in vitro cell studies, whereas less information is available on how these mechanisms play out in in vivo cells.

The mitochondrial life cycle begins with a phenomenon known as mitochondrial biogenesis (mitochondrion-genesis) and ends with the degradation of damaged or supernumerary organelles, through a cellular phenomenon called mitophagy. The mitochondrial renewal process, that is a good balance between mitochondrion-genesis and mitophagy, is essential to optimize the functionality of cellular respiration. The newly formed mitochondria have a high capacity for energy production, in contrast, aged mitochondria have reduced metabolic capacity [18].

Mitochondria have a variable life which is tissue and cell type-specific. Moreover, published data on mitochondrial turnover are scarce and differ from each other by up to one order of magnitude. These differences are largely methodological. By measuring the rate of loss of radioactivity of mitochondria labelled with [^35^S] methionine and [^14^C] acetate, Fletcher and Sanadi [19] first reported that rat liver mitochondria turn over with a half-life of about 10 days. They also found that protein and lipid fractions of rat liver mitochondria had almost identical turnover rates and concluded that liver mitochondria turn over as discrete units. Subsequently, other researchers reported data indicating that the conclusion that the mitochondria turn over as a unit could not be extended to other tissues [20,21,22,23]. Subsequent works from several laboratories suggested that, although the inner membrane and associated matrix, which accounts for most of the particle mass, appears to turn over as a unit, a portion of the mitochondrion, particularly the outer membrane and several of inducible enzymes, may turn over independently [24,25,26,27]. Thus, bulk-labelling turnover studies would be expected to reflect the stability of this organelle. It is worth noting that re-utilization of the radioactively labelled precursor in pulse-chase assays is the most likely cause of significant overestimation of mitochondrial turnover rates. Thus, determinations using a minimally reutilized amino acid precursor ([^14^C] bicarbonate) indicated that the liver mitochondrial half-life is 3.8 days [27]. More recently, estimating the extent of label re-utilization with a dynamic mathematical model, it has been reported that actual liver mitochondrial half-life is only 1.83 days [28].

During their lifetime, the mitochondria are also subjected to numerous cycles of fission and fusion. At the end of the 80s, microscopy studies [29] showed that mitochondria are very dynamic organelles and can build up a dense network of intracellular connections, although they appear as single entities. The two opposite and balanced activities, fusion and fission, give life to the mitochondrial compartment of the cell, and this dynamic arrangement of the mitochondria allows the cells to respond to the different changes in physiological conditions. A displacement of the mitochondrial balance towards fusion favors the formation of interconnected mitochondria, while if it moves towards fission many mitochondrial fragments are formed. The widespread network of mitochondria generated by fusion is advantageous in cells with an active metabolism, contributing to energy dissipation. In contrast, in quiescent cells, the mitochondria are present in the form of numerous, small, and distinct spheres or rods [30].

## 4. Mitochondrial Biogenesis

The word biogenesis has been used to describe both the formation of new mitochondria during the life cycle of a cell and their phylogenesis [31]. As a comprehension of the evolutionary origin is a prerequisite for understanding any biological structure or process, we address the interested readers to thoughtful reviews on this topic [32,33,34]. Here we will focus our attention only on the problem of mitochondrial formation.

Research on such a topic was one of the youngest areas of “mitochondriology”. This is not surprising since the problem of mitochondria formation could only be addressed after the morphology, composition, and function of the organelles had become quite known.

For a long time, the problem of the formation of mitochondria was unclear. A clue for its solution came from the discovery that the mitochondrial population is heterogeneous regarding its characteristics because it consists of fractions with different properties [35]. Gear [36,37], working with regenerating rat liver, separated mitochondria into heavy, light, and fluffy fractions and concluded from his studies that the light fraction may have a precursor role whereas the fluffy particles may represent a mixture of regenerating and degenerating mitochondria with microsomes. The subsequent observation that the light mitochondrial fraction lacked the ability of coupled phosphorylation supported the idea that the light mitochondria could be immature forms in the process of developing into stable heavy mitochondrial structures [38].

Thus, available data indicated that the formation of functional mitochondria depends on two different processes, organelle proliferation and organelle differentiation. Organelle proliferation is an expansion of the mitochondrial mass, while mitochondrial differentiation can be defined as an increase in the functional capabilities of pre-existing mitochondria to acquire the ultrastructure and functional features of mature mitochondria. However, studies dealing with mitochondrial heterogeneity did not indicate the mode of formation of the light mitochondria.

Over time, mitochondrial biogenesis was attributed to three different processes: (i) de novo synthesis from cytoplasmic sub-microscopic precursors; (ii) formation from other cellular membranes; (iii) growth and division of pre-existing mitochondria.

In the 40s, experiments performed on sea urchin eggs [39] and mouse kidney tubules whose pre-existing mitochondria had been removed or destroyed [40] seemed to indicate that these cells could regenerate their mitochondria, suggesting that mitochondria were derived from other cellular structures. In contrast, optical and electronic microscopy studies performed in the 1950s and early 1960s seemed to show that mitochondria were formed from other cellular membranes, in particular, the nuclear outer membrane [41]. Moreover, it was reported that anaerobically grown yeast cells which were devoid of mitochondria, could reform them following oxygen exposure [42,43].

The idea of a de novo generation of mitochondria lost credibility in the late 60s following the discovery that mitochondria of anaerobically grown yeast cells were not lost but were dedifferentiated into respiration-deficient pro-mitochondria which are hardly detectable by conventional electron microscopy [44,45]. On the other hand, an attack on the credibility of the de novo generation of mitochondria also derived from the discovery of the mitochondrial DNA. This was first detected in abnormal chicken fibroblasts by Frederic and Chèvremont [46]. Late in 1963, electron micrographs were published which indicated that the mitochondria of chick embryo cells contained thread-like structures that could be digested by DNAse, but not by RNAse [47,48].

It is interesting to note that clues of the existence of a mitochondrial genetic system had already been obtained when Ephrussi [49] and Mitchell and Mitchell [50] had found that the properties of certain respiration deficient *Saccharomyces cerevisiae* and *Neurospora crassa* mutants were not inherited according to Mendelian laws. Although earlier studies had suggested that hereditary determinants may reside outside the nucleus [51], the properties of these microbial mutants suggested that extrachromosomal genetic factors might reside in mitochondria. However, interest in these mutants remained confined to a few specialized laboratories and research on mitochondrial formation was unfortunately delayed.

Around the same time, it was discovered that isolated rat liver mitochondria could incorporate labelled amino acids into protein [52], and that the antibiotic chloramphenicol inhibited protein synthesis by isolated mitochondria but not by microsomes [53] and blocked the formation of respiring mitochondria in intact yeast cells [54]. These results demonstrated that mitochondrial protein synthesis was a biological reality and that it was necessary for the biogenesis of respiring mitochondria. As yeast and animal cells grown in the presence of chloramphenicol proved to be deficient in cytochrome c oxidase and cytochrome b [55], it seemed likely that these mitochondrial components were made at least partly within mitochondria. This idea was supported by the isolation of components of the machinery needed to replicate and express the genes contained in mitochondrial DNA [56,57].

With the advancement of the available techniques, it was also possible to demonstrate that mitochondria synthesize three (out of the seven) subunits of cytochrome c oxidase [58], at least two (out of the approximately 10) subunits of the oligomycin-sensitive ATPase complex [59], and one (out of the six to nine) subunits of the cytochrome b-c complex [60,61]. In 1981, when the human mtDNA was sequenced [62], it was established that just 13 proteins are encoded in it, a small fraction of those known to be mitochondrially located. The main conclusion from these studies that mitochondria do not synthesize complete enzymes, but only some subunits of oligomeric enzymes, is still valid today. The remaining subunits of these enzymes and the hundreds of other proteins that are found in mitochondria are encoded outside the mitochondria in nuclear, chromosomal DNA, are constructed in the cytoplasm just like all other chromosomally encoded proteins and are then imported into mitochondria. Therefore, mitochondrial genesis requires the coordinated expression of multiple genes encoded in two physically separate genomes.

All proteins encoded in mitochondria are essential components of the respiratory complexes whose assembly requires coordination with the expression and import of nuclear-encoded proteins. The inability to coordinate this process can lead to unassociated proteins that are susceptible to misfolding and aggregation.

Approximately when mtDNA was identified, a related discovery was made, that favored the idea that for mitochondrial biogenesis pre-existing mitochondria may grow and divide. In fact, in his classical experiments, Luck [63,64] excluded the possibility of de novo formation of mitochondria and did not favor the hypothesis of structural mitochondrial precursors. Conversely, his data suggested that in *Neurospora* growing in a medium containing radioactive choline the mitochondrial mass was increased by a continuous process of addition of new choline-containing lipid units to the existing structural framework. The number of mitochondria in the population of *Neurospora* transferred to the unlabeled medium was increased by the division of mitochondria. The division process distributed the label at random so that pre-existing mitochondrial membranous material was transmitted uniformly or nearly uniformly to all progeny.

Unfortunately, is hard to pinpoint the moment when a new mitochondrion is “born”. This is because mitochondrial biogenesis is a drawn-out process with many steps, which in principle can occur independently and be balanced in different ways depending on the cell’s metabolic needs.

It is apparent that whenever a cell divides, its mitochondria must also double their size and divide providing daughter cells with a normal complement of mitochondria. Mitochondrial mass increases from the onset of S-phase through M-phase [65]. In the human, the copy number of mtDNA varies with the cell type and is usually maintained within a range [66]. Thus, under normal physiological conditions, mtDNA molecules double in every cell cycle, which is required if each daughter cell is to maintain a constant amount of mtDNA. In addition, it has been shown that the mtDNA replication, occurs predominantly in the late S and G2, but may occur throughout the cell cycle [67]. Hence, mtDNA replication does not occur concurrently with the growth and division of the organelles [68] and may not be coupled with mitochondrial proliferation.

On the other hand, there are instances in which mitochondrial biogenesis and mtDNA are not produced in association with cell division.

Many body cells, such as nerve and muscle cells, (so-called postmitotic) do not undergo division, but even in them, there is a turnover of mtDNA. Even though in theory, all the proteins in a mitochondrion could be recycled individually without the mitochondrion dividing, if the mtDNA is recycled, the mitochondria themselves must divide. This means that mitochondria must also be destroyed in postmitotic cells to prevent cells from filling up with mitochondria.

Furthermore, the number of mitochondria, as well as the number of copies of mtDNA, in a cell can change according to different energy needs and different physiological and environmental conditions as demonstrated by the proliferation of muscle mitochondria during cold exposure [69] and physical training [70]. However, the regulation of the number of copies of mtDNA and the number of mitochondria in different physiological and developmental conditions is not yet fully clarified.

Controlling the biogenesis of mitochondria and maintaining mtDNA is a complex biological process. However, it is known that all proteins involved in the replication, transcription, and translation of mtDNA in mitochondria [68], as well as several protein factors involved in the mitochondrial biogenesis and in maintaining mtDNA copy numbers [71], are encoded by nuclear genes.

### 4.1. Mediators of Increase in Mitochondrial Mass

**Figure 1:**
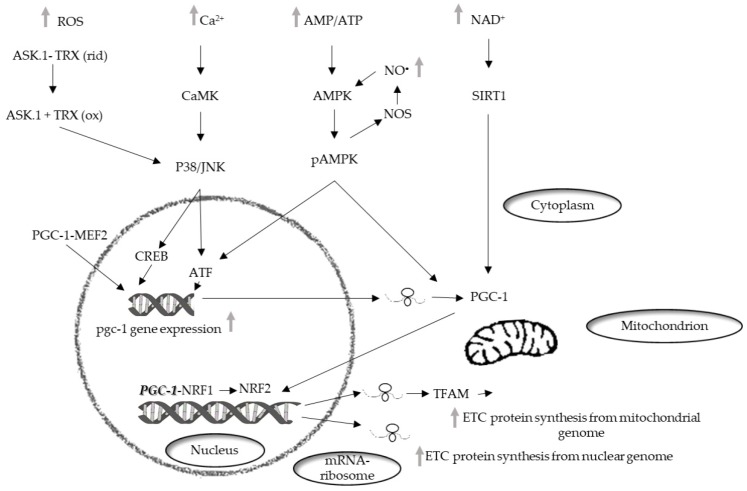
Schematic representation of the signaling pathways that mediate the mitochondrial biogenesis. PGC-1, peroxisome proliferator–activated receptor coactivator 1; NRF-1, nuclear respiratory factor 1; NRF-2, nuclear respiratory factor 2; MEF2, myocyte enhancer factor-2; cAMP, cyclic adenosine monophosphate; CREB, cAMP response element-binding protein; AMPK, AMP-activated protein kinase; PKA, protein kinase A; NO•, nitric oxide; NOS, nitric oxide synthase; CAMK, Ca^2+^/calmodulin-dependent protein kinase; p38, p38 mitogen-activated protein kinases; JNK, c-Jun N-terminal kinase; ASK-1, ETC, electron transport chain.

Mitochondrial biogenesis involves a phase which necessitates the coordinated expression of both mitochondrial and nuclear genomes and leads to an increase of the mass of mitochondria [72] (Bouchez, 2019). In mammalian cells, the main actors of mitochondrial biogenesis are the nuclear respiratory factors (NRFs), NRF1, in particular [73]. The NRFs are known to be involved in the transcription of several mitochondrial genes in particular genes encoding subunits of the mitochondrial respiratory chain complexes. Furthermore, these factors regulate the transcription of mitochondrial transcription factor A (TFAM or mtTFA), which regulates the replication and transcription of the mitochondrial genome [74] (Figure 1).

#### 4.1.1. Role of PGC-1

The transcriptional family of peroxisome proliferator-activated receptor γ (PPARγ) coactivator-1 (PGC-1) is also involved in the regulation of mitochondrial biogenesis. The components of the family, PGC-1α, PGC-1-related coactivators (PRC), and PGC-1β, can interact with other transcription factors involved in the expression of mitochondrial proteins. PGC-1α was first identified in brown adipose tissue [75] and was subsequently found in other mitochondria-rich tissues, such as skeletal and cardiac muscle, kidney, liver, and brain [76], in which it affects several metabolic processes [77]. PGC-1α interacts with transcription factors such as NRF1 and 2 and regulates their expression. Moreover, interacting with myocyte enhancer factor 2 (MEF2), its own upregulation [78].

Factors belonging to the MAPK family are also involved in mitochondrial biogenesis regulated by PGC-1. The MAPK family, which is involved in the conversion of cell signals into cellular responses, contribute to the regulation of life-and-death decisions elicited by several stress signals including ROS [79]. MAPKs control numerous pathways of cellular signaling activating or deactivating regulatory proteins through phosphorylation [80].

In mammalian cells, four groups of MAPKs have been characterized such as the c-Jun N-terminal kinases (JNK), the extracellular signal-related kinases (ERK1/2), the p38 kinase (p38), and the big MAP kinase 1 (BMK1/ERK5). Cytokines, growth factors, and cellular stress can stimulate the MAPKs although their relative activation and the specific cellular response evoked depend on the type of stimulus [81].

All MAPKS are proline-directed serine/threonine kinases and are activated through a cascade of phosphorylation events, referred to as the MAP kinase module, in which a MAP kinase kinase kinase (MAPKKK) phosphorylates and activates a MAP kinase kinase (MAPKK), which in turn phosphorylates and activates a MAPK [80,82].

In skeletal muscle, MEF2 can activate p38 [83], which, in turn, can regulate the expression of PGC-1α by activating transcription factor 2 (ATF2) [84]. Another factor inducing PGC-1α expression is Ca^2+^/calmodulin-dependent protein kinase (CaMK). It is activated by an increase in the intracellular Ca^2+^ concentration in skeletal muscle, and induces the expression of PGC-1α via cAMP response element-binding protein (CREB) [85].

PGC-1α regulation is not only due to variation in its expression but is also caused by its covalent modifications including phosphorylation, acetylation, methylation, and ubiquitination [86].

AMP-activated protein kinase (AMPK) is considered the major factor of PGC-1α post-translation modification for a wide range of tissues. PGC-1α phosphorylation is only one of the mechanisms of mitochondrial biogenesis activation via AMPK [87]. Indeed, AMPK can increase the level of NAD^+^, which results in silent information regulator 1 (SIRT1) phosphorylation. Activated SIRT1 acts as another upstream regulator of PGC-1*α* [88], which activates PGC-1*α* by deacetylation, thereby promoting mitochondrial biogenesis [89]. p38 MAPK also, besides regulating the expression of PGC-1, can activate it by phosphorylation [90] and in vitro experiments have shown that PGC-1α phosphorylation by p38 MAPK and AMPK produces a more active protein [91].

The events initiating the PGC-1 activation and induction of the biogenesis program have not been fully deciphered. Mitochondrial biogenesis and mitochondrial mass can be modulated through several stimuli and cellular pathways, including hormones, such as thyroid hormones [92] and catecholamines [93], inflammatory signaling [94], as well as calcium signaling [95].

Despite these problems, the current knowledge of the role played by PGC-1 in mitochondrial biogenesis has allowed the development of therapies to treat several diseases through triggering mitochondrial biogenesis by pharmacological modulation of PGC-1.

As mitochondria are involved in numerous processes necessary for the maintenance of cellular physiology and homeostasis, mitochondrial dysfunction is a prominent feature of a wide spectrum of human diseases [96]. Mitochondrial dysfunction may result from genetic mutations in mtDNA and/or nuclear genome. Some mitochondrial diseases arise from specific mutations in nuclear genes, which are mainly involved in the assembly and function of respiratory enzyme complexes, biogenesis of mitochondria, and maintenance and replication of mtDNA [97]. mtDNA mutation causes impairment of the OXPHOS system or other defects in the energy metabolism of mitochondria and plays a pivotal role in the pathophysiology of diseases such as neurodegenerative diseases and cancers [97].

Mitochondrial dysfunction has also been implicated as an integral part of the pathology of non-genetic metabolic disorders, such as obesity and metabolic syndrome [98].

The current treatment of mitochondrial diseases varies considerably. Clinical goals of mitochondrial disease therapy are to increase the energy yield and reduce ROS production and thus improve the symptoms of the disease. Although there are several treatment strategies for OXPHOS disorders and mitochondrial dysfunction, the main therapies have focused on the way to increase mitochondrial biogenesis and/or energy production, with an attempt to halt disease progression and improve the function of affected tissues of patients. Moreover, accumulating experimental evidence supports the strategy to treat mitochondrial diseases by enhancement of mitochondrial biogenesis and metabolic activity through pharmaceutical modulation of PGC-1 [99].

Pharmacological activation of mitochondrial biogenesis can be achieved with a large range of compounds [100]. Among these, the pan-PPAR (α, β/δ, γ) agonist bezafibrate stimulates the heart and skeletal muscle PGC-1α expression in mice in a PPARδ-dependent manner [101]. In vitro experiments have shown that bezafibrate treatment is able to upregulate respiratory capacity and OXPHOS function in fibroblasts and myoblasts from OXPHOS patients [102]. Moreover, dietary bezafibrate administration has been shown to result in an increase in PGC-1 expression, induction of mitochondrial biogenesis in skeletal muscle, and prolonged survival of ΔCox10 mice [103]. Interestingly, bezafibrate is a relatively safe compound and is widely used by humans for the treatment of dyslipidemia because its damaging effects on the liver in mice are not observed in humans [104].

#### 4.1.2. Role of Nrf2

We should emphasize that not only the number of mitochondria is important, but also their functional state, which depends on mitochondrial biogenesis and dynamics, including fission/fusion and mitophagy. The coordination between these processes is controlled by several mutually regulated signaling cascades, one of the most important being the Nrf2/ARE cascade [105].

Despite a growing interest in this signaling pathway, there are only a few reviews on the key role of Nrf2 in mitochondrial biogenesis and its interactions with the more studied “master regulator of mitochondrial biogenesis” PGC-1α [106].

Reference to Nrf2 (NF-E2-related factor 2) first appeared in the scientific literature in 1994 [107] and has subsequently emerged as a key modulator of the cell’s primary defense mechanism, countering many harmful environmental toxicants and carcinogens [108]. Furthermore, Nrf2 is upregulated by some longevity-promoting interventions and may play a role in regulating species longevity [109,110].

Nrf2 is a basic region-leucine zipper-type transcription factor belonging to the cap ‘n’ collar (CNC) family [107,111] that tightly interacts with a cytosolic inhibitor (INrf2), also known as Keap1 (Kelch-like ECH-associated protein 1).

Under unstressed conditions, Keap1 constitutively inhibits Nrf2 signaling by acting as an adaptor subunit for cullin-3-based ubiquitin E3 ligase [112]. This E3 ligase complex efficiently ubiquitinates Nrf2, leading to its rapid proteasomal degradation [113].

In the presence of agents that oxidize SH groups in cysteine residues [114], Nrf2 degradation is hindered because of a conformational change in Keap1 that results in loss of its ubiquitin ligase activity. Nrf2 is released from Keap1 and free Nrf2, phosphorylated by several protein kinases including protein kinase C (PKC) [115], translocates to the nucleus. Here, it forms a heterodimer with the small protein Maf which in turn facilitates the binding of Nrf2 to ARE in the promoter region of Nrf2-regulated genes allowing for coordinate induction of multiple cellular defense enzymes [116,117,118].

Another well-described Nrf2 repressor is glycogen synthase kinase 3 β (GSK3β), a multifunctional serine/threonine kinase, which plays a major role in various signaling pathways [119]. Unlike most protein kinases, GSK3β is active under non-stress conditions and can phosphorylate Nrf2, thereby suppressing its translocation to the nucleus. However, GSK3β-induced suppression of Nrf2 can be abolished by protein kinase B (Akt/PKB) that inhibits GSK3β [120]. It is also reported that nuclear import of Nrf2, which, from the time of exposure to the stabilization, takes roughly two hours [121], is followed by activation of a delayed mechanism involving GSK3β. This mechanism controls switching off Nrf2 activation of gene expression. GSK3β phosphorylates Fyn, a tyrosine kinase at unknown threonine residue(s) leading to nuclear localization of Fyn [122]. Fyn phosphorylates Nrf2 tyrosine 568 resulting in nuclear export of Nrf2, binding with INrf2 and degradation of Nrf2 [123].

The involvement of the Nrf2/ARE signaling cascade in mitochondrial biogenesis is a relatively recent idea, as the role of Nrf2 in the activation of mitochondrial biogenesis was suggested for the first time only in 2008 [124]. The discovery of four AREs in the NRF1 gene promoter capable of Nrf2 binding was the stimulus for the development of this field of research.

The production of H_2_O_2_ stimulated by carbon monoxide causes oxidation of phosphatase and tensin homolog (PTEN), which results in Akt/PKB activation. Akt, in turn, phosphorylates and inactivates GSK3β, thus promoting Nrf2 translocation to the nucleus where Nrf2 binds to the NRF1 promoter AREs. NRF1 activates TFAM, which regulates the mtDNA replication [124].

The demonstration of the key role of Nrf2 in mitochondrial biogenesis was subsequently obtained in studies using Nrf2-deficient animal models which showed that, unlike the wild-type animals, the amounts of mitochondrial markers in the Nrf2−/− mutants were not increased by Nrf2 activators [125,126].

Nrf2 translocation to the nucleus was found to be strictly regulated by the activity of AMPK which phosphorylates Nrf2 at Ser50, which results in GSK3β inactivation, both processes being necessary for Nrf2 nuclear translocation [127].

However, the evidence is available that Nrf2 is controlled by PGC-1α, which regulates the antioxidant genes through Nrf2 activation [128]. Indeed, downregulation of PGC-1α expression results in almost complete inhibition of Nrf2 binding to the gene codifying the glutamate-cysteine ligase catalytic subunit (GCLC) and decreases the content of SOD2 and glutamate-cysteine ligase enzymes. Furthermore, PGC-1α knockout results in dysregulation of the mitochondrial biogenesis dependent on Nrf2 [129]. It was demonstrated that PGC-1α activates Nrf2 through p38-mediated inhibition of GSK3β [130]. Thus, the PGC-1α/p38/GSK3β/Nrf2 cascade is most likely the pathway of connection between the two coregulators and mtDNA transcription.

It is also possible that Nrf2 and PGC-1α form the feedback loop, i.e., Nrf2 directly influences PGC-1α expression. There are data supporting such an idea: (i) the PGC-1α gene promoter contains two AREs: −1723 (50-TC TTGATGACATTGCTTCTG-30) and −226 (50-CTGATT TGATGGAGCTACTT-30) [131]; (ii) the siRNA-mediated suppression of Nrf2, as well as Nrf2 knockout, inhibit mitochondrial biogenesis and downregulate PGC-1α expression in hepatocytes [132] and skeletal muscles [133].

There is evidence that both signaling cascades could be activated simultaneously. Nrf2 and PGC-1α could be simultaneously activated via the Erk1/2 signaling cascade. Erk1/2 activates both Nrf2 and PGC-1α via phosphorylation of the serine/threonine kinase LKB1 (liver kinase B1), a known tumor suppressor, which in turn phosphorylates AMPK [134].

### 4.2. Role of ROS in Mitochondrial Biogenesis

It has been shown that in mammalian cells mitochondrial biogenesis is strongly dependent on cell ROS production, whose increase can result in either a decrease or an increase in mitochondrial biogenesis. It was showed that oxidative stress, induced by indoxyl sulphate in the endothelial cells of the human umbilical vein, leads to a decrease in mitochondrial biogenesis, which can be countered by treatment with N-acetylcysteine (NAC) [135] a precursor of GSH which is also able to directly reduce disulphide bridges [136]. Thus, the polyphenol resveratrol protects mouse T cells against apoptosis induced by the high-fat diet by reducing oxidative stress and stimulating mitochondrial biogenesis [137].

In contrast, other reports suggest that oxidative stress is involved in the increase of mitochondrial content and mtDNA copy number in human and animal cells. Increases in the mitochondrial mass and the mtDNA copy number of human cells are induced by moderate concentrations of H_2_O_2_ and buthionine sulphoximine, which lowers tissue glutathione concentrations [138]. H_2_O_2_ treatment and replicative senescence of human cells increase the mRNA levels of many nuclear DNA-encoded proteins involved in mitochondrial biogenesis [139]. NRF-1 activity and mtTFA gene expression are increased and mtDNA replication and cell proliferation are stimulated by oxidative stress induced in rat liver by injection of lipopolysaccharides (LPS) [140]. Expression of PGC-1α and components of the mitochondrial antioxidant defense system, such as SOD1, SOD2, catalase, or glutathione peroxidase (GPX), is enhanced by oxidative stress in neural cells [141].

The above conflicting results can be explained admitting that the oxidative stress effects depend on the severity and duration of the stress so that acute/mild stress stimulates PGC-1α expression and mitochondrial biogenesis whereas severe/permanent stress has opposite effects.

The discrepancy was also found in the effects of antioxidant supplementation on mitochondrial biogenesis induced by exercise training. Thus, antioxidant treatment of rats was reported to impair muscle mitochondrial biogenesis and prevent increases in mitochondrial biogenesis markers [142,143]. In contrast, antioxidant treatment of mice was shown to suppress training-induced increases in mitochondrial biogenesis markers even though this did not affect exercise training-induced increases in mitochondrial mass [144,145]. It is possible that the discrepant results are due to differences in parameters of training protocols and factors such as duration and timing of supplementation, type of antioxidant, which affect the amount of ROS produced during the single sessions of training and the extent to which antioxidants scavenge ROS.

The observation that treatment of cultured muscle myotubes with exogenous H_2_O_2_ activates AMPK and increases Pgc-1α expression [146] suggested that H_2_O_2_ can promote Pgc-1α expression through AMPK. Subsequently, it was demonstrated that the phosphorylation and activation of AMPK due to H_2_O_2_ treatment was likely mediated via the ROS-induced decrease in ATP levels since NAC prevented the decline in ATP levels in cells treated with H_2_O_2_ and coincidentally attenuated AMPK activation [147]. More recently it has been found that ROS can activate AMPK indirectly [148]. Notwithstanding this, the idea that the effects of ROS on Pgc-1 expression are mediated indirectly, via AMPK activation, was confirmed by the observation that NAC also inhibited ROS-associated Pgc-1α upregulation [149].

It is worth noting that, notwithstanding that the results obtained administering exogenous H_2_O_2_ are questionable [150], PGC-1α expression in muscle can be regulated by a variety of stimuli associated with muscular exercise, which, however, seem to be dependent on ROS production. Thus, the finding that the human PGC-1α promoter contains a binding site for NF-κB suggests that the expression of PGC-1α may also be regulated by NF-κB [147]. NF-κB is one of the most commonly investigated redox-sensitive transcription factors. The NF-κB/Rel transcription factors are normally sequestered in the cytoplasm in an inactive state, linked to the IκBα inhibitory protein. NF-κB is activated by several stimuli, including H_2_O_2_, by the phosphorylation of IκBα at Ser-32 and -36 by IκB kinase (IKK). Phosphorylation of IκBα results in its dissociation from NF-κB and subsequent proteasomal degradation. NF-κB, once free, migrated into the nucleus where it binds to the corresponding DNA sequence of the target genes [149]. In muscle cells, ROS such as H_2_O_2_ are able to induce degradation of the inhibitory IκB protein subunits bound to NF-κB subunits, leading to the rapid migration of NF-kB to the nucleus and activation of the transcription of specific genes [151].

Analysis of the human PGC-1α promoter has revealed a variety of consensus transcription binding sites to other transcription factors. These include specificity protein 1 (SP1), CREB, CREB related family member, ATF2, forkhead transcription factor (FKHR), p53, E Box binding proteins, GATA, and MEF2 [152]. Many of these transcription factors have been shown to be ROS-sensitive, which indicates numerous potential possibilities for redox control of PGC-1α expression.

The three best-characterized MAPK subfamilies, JNK, p38 MAPK, and ERK, are activated by oxidative stress and could potentially be involved in pathways affecting the breakdown of muscle proteins or loss of nuclei via myonuclear apoptosis [153,154]. It has also been shown that H_2_O_2_ can elicit the activation of ERK, JNK, and p38 MAPK in skeletal myoblasts in a dose and time-dependent manner [155].

Activation of MAKs seems to depend on the apoptosis signal-regulating kinase-1 (ASK-1), a member of the MAPKKK family. ASK-1 is modulated by redox mechanisms and activates both p38 and JNK pathways [156]. In unstimulated conditions, ASK-1 binds to the repressor protein thioredoxin (Trx), a ubiquitously expressed redox regulatory protein, so that its kinase activity is inhibited. The binding of Trx to ASK-1 requires the presence of a reduced form of an intramolecular disulfide bridge between two cysteine residues in the catalytic site of Trx. This protein, after its oxidation by ROS molecules such as H_2_O_2_, dissociates from and liberates ASK-1, which is then activated by the formation of an oligomeric complex and threonine autophosphorylation [156].

RNS, particularly NO^•^, may also be involved in the regulation of PGC-1α. The idea that NO^•^ mediates the upregulation of PGC-1a, thus modulating mitochondrial function and biogenesis, is supported by the evidence that low levels of NO^•^ induce mitochondrial biogenesis, PGC-1α and GLUT4 expression in cultured muscle cells [157] and that genetic deletion of NOS or their pharmacological inhibition prevents PGC-1a induction that is triggered by endurance exercise [158].

It has also been observed that administration to humans of inorganic nitrate (which can be converted into NO^•^ in the body) significantly improves energy metabolism during exercise [159].

Reports showing that NOS activity is involved in mitochondrial biogenesis induced by AMPK and CaMK and PGC-1 α expression in L6 myotubes [160] and that AMPK phosphorylates and activates both eNOS and nNOS [161], led to the proposition that there is a positive feedback loop between NO^•^ production and AMPK activity in skeletal muscle [162]. The evidence that NO^•^ production promotes PGC-1 α expression via NO-mediated activation of AMPK (i.e., AMPKα1 isoform) demonstrated that the proposed model of synergistic interaction between AMPK and NOS is crucial to maintain metabolic function in skeletal muscle cells [162]. Moreover, it suggested that both ROS and RNS can contribute to PGC-1α expression via a common signaling pathway (i.e., AMPK activation).

Finally, it has been hypothesized that the expression of genes codifying for subunits of the mitochondrial electron can respond to physical environmental change by means of a direct control exerted by the change in the redox state of the corresponding gene product. Thus, it has been suggested that to preserve function, the redox regulatory system is retained within its original membrane-bound compartment. The colocation of gene and gene product for redox regulation of gene expression (CoRR) is a hypothesis in agreement with the results of a variety of experiments designed to test it and which seem to have no other satisfactory explanation [163].

## 5. Dynamics of Mitochondria

**Figure 2:**
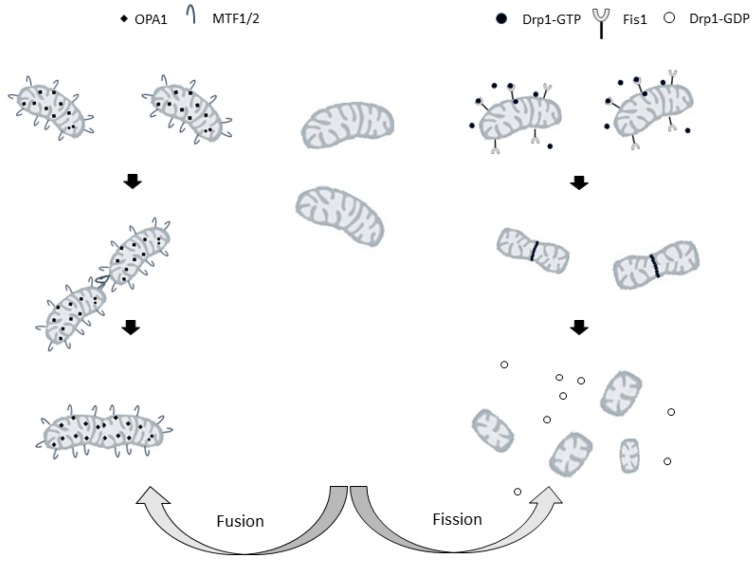
Representation of the mitochondrial fusion (on the left) and fission (on the right) processes. Drp1: cytosolic dynamin-related protein 1; FIS1: mitochondrial fission protein 1. MFN1/2: mitofusin 1 or 2; OPA1 optic atrophy factor 1.

Mitochondria are organelles which exhibit a heterogeneous structure because of the balance between fusion and fission of mitochondrial membranes [164] (Figure 2).

In the 1980s live-cell microscopy studies showed that mitochondria are highly dynamic organelles that are organized in large interconnected networks in the cell [164]. In many types of eukaryotic cells, mitochondria are continuously moving along cytoskeletal tracks and undergo frequent fusions and divisions. Such activities control the morphology and intracellular distribution of mitochondria in the cell.

The evidence that changes in mitochondrial shape are linked to neurodegeneration, calcium signaling, lifespan, and cell death indicate that the morphology and the regulation of mitochondrial shape are crucial for cellular physiology. Moreover, morphology and function of mitochondria are closely linked, so that dysfunctional mitochondria arise from the loss of fusion or division activity [165]. The importance of mitochondrial fusion can be explained by the need for an exchange of the contents of intermembrane space and matrix between mitochondria, so that defects and transient stresses may be partially buffered. For yet misunderstood reasons, mtDNA copy number is drastically affected by severe impairment of mitochondrial fusion. In budding yeast, mtDNA maintenance depends on mitochondrial fusion [166], whereas in mammals, loss of fusion results in depletion but not a total loss of mtDNA [167].

On the other hand, the mitochondrial division should create organelles of the appropriate size for transport along with actin or microtubule networks [165]. The balance between fission and fusion on which mitochondrial shape depends is controlled by multiple proteins that mediate the remodeling of the outer and inner mitochondrial membranes [18].

### 5.1. Fission

Scission from the mitochondrial network is an important process that either contributes to biogenesis (i.e., adjusting the number of mitochondria to an increase in mitochondrial mass, thus ensuring that the single mitochondria have the same mass) or fragmentation (i.e., generating a larger number of smaller mitochondria).

In mammalian cells, important roles in regulating mitochondrial division are played by cytosolic dynamin-related protein 1, Drp1 [168] and mitochondrial fission protein 1 (FIS1), a small protein anchored in the mitochondrial outer membrane (MOM) [169]. Drp1 is a GTPase that must be activated and recruited to mitochondria to induce mitochondrial fission [170]. Its activity is regulated by post-translational modifications [171], and, in particular, the phosphorylation status of Drp1 determines its localization and its effect on the mitochondrial structure. Phosphorylation at Ser-616 activates Drp1, whereas phosphorylation at Ser-637 inhibits Drp1, preventing the protein translocation to the sites of mitochondrial division and, therefore, inhibiting organelle fission [172].

Upon activation, molecules of Drp1 translocate to the mitochondria, where they promote mitochondrial fission by forming a ring-like structure around the organelle. The cross-bridging of the GTPase domains of adjacent Drp1 proteins leads to GTP hydrolysis, constriction, and the final cut of mitochondria [173].

Drp1 inhibition increases the length and interconnectivity of mitochondrial tubules, thereby inhibiting the fission process and preventing cell death [174].

It is worth noting that recent evidence suggests that another MOM anchored protein, the mitochondrial fission factor (MFF), is essential for DRP1 recruitment [175]. Moreover, it has recently been proposed that DRP1 is not enough to execute mitochondrial fission and that another GTPase, dynamin- 2 (DNM2, also known as DYN2), is an essential component of the mitochondrial division machinery [176].

### 5.2. Fusion

In fusion, the membranes of two mitochondria are joined to form a single mitochondrion. Three dynamin-related GTPases, mitofusins 1 (MFN1) and 2 (MFN2), and optic atrophy factor 1 (OPA1) are the main players in this process. The fusion of both membranes is necessary to merge two mitochondria. In mammals, the principal regulators of the fusion of mitochondrial outer and inner membranes are mitofusins and OPA-1, respectively [177].

Due to the crucial role of each mitofusin in mitochondrial fusion, single knockouts of either Mfn1 or Mfn2 remarkably reduce the rates of mitochondrial fusion [178] increasing mitochondrial fragmentation. However, the shapes and sizes of the resulting mitochondria depend on which mitofusin is knocked down [178]. Indeed, Mfn1 depletion leads to the formation of small vesicular mitochondria dispersed in the cell, whereas Mfn2 lack results in the formation of larger vesicular mitochondria concentrated around the nucleus [178]. It is apparent that each mitofusin has a specialized role in the fusion process [179] and it has been shown that GTP hydrolysis-dependent mitochondrial tethering specifically requires Mfn1, whereas Mfn2 is less efficient in this fusion step [179].

The two mitofusins also play roles in cell physiology. MFN2 is the more versatile and participates in cell metabolism, tethering the endoplasmic reticulum to mitochondria, and cell proliferation [180]. Furthermore, MFN2 gene mutations are associated with a peripheral motor neuropathy (Charcot–Marie–Tooth syndrome type 2A) [181].

OPA1, the other dynamin-like GTPase, is a key pro-fusion player that is anchored to the mitochondrial inner membrane [182]. It is mutated in the autosomal-dominant optic atrophy (ADOA) [183], a hereditary neuropathy leading to the loss of fibers of the optic nerve. Moreover, spastic paraplegia, another peripheral neuropathology, is associated with mutations of paraplegin, an m-AAA-protease that is required for mitochondrial processing of OPA1 [184].

Complex post-transcriptional mechanisms tightly regulated both OPA1 and the activities of other mitochondria-shaping proteins including proteolytic processing [185,186]. Other than its fusogenic activity OPA1 shows other functions: different forms of OPA1 aggregates to form heterocomplexes that control the structure of the cristae and the release of cytochrome *c* regulates apoptosis [187,188].

### 5.3. Role of ROS in Mitochondrial Dynamics

The activation of either salvaging or pro-death pathways is accompanied by the collapse of the reticular form of mitochondria into fragments. The fragments are dysfunctional mitochondria that are selectively targeted for mitophagy promoting the survival of the cell [189] or, when the oxidative stress is high, determine the onset of the apoptotic pathway [190]. In both apoptotic and mitophagic stress response mitochondrial fission is involved, which seems to be triggered by mitochondrial ROS [191,192].

In 2004, Skulachev and collaborators, in their studies on pig kidney cell culture whose mitochondria was stained by penetrating fluorescent cation ethyl rhodamine, found that the treatment with rotenone, a respiratory complex I inhibitor, resulted in complete disappearance of mitochondrial filaments. Instead, numerous grain-like mitochondria appeared due to the decomposition of extended mitochondrial profiles (the thread-grain transition) [193]. It was also suggested that thread-grain transition might be useful for the cell in response to the appearance of ROS.

It is known that ROS treatment of mitochondria in vitro results in the opening of so-called permeability transition pore (PTP) in the inner mitochondrial membrane [194] and that ROS when added to cell culture, induce apoptosis [195,196].

It was suggested that the ROS-induced PTP opening represents a mechanism of the suicide of the mitochondrion [197]. A mitochondrion with opened PTP fails to maintain membrane potential and, hence, cannot import mitochondrial proteins synthesized in the cytosol. Such a mitochondrion cannot be repaired and must perish. This effect called ‘mitoptosis’ [198] can purify the mitochondrial population in the cell from those organelles that overproduce toxic O_2_ derivatives as ROS [199]. Thus, if some part of the mitochondrial reticulum becomes a ROS-super producer, the situation can be improved by decomposition of the reticulum to small isolated mitochondria and mitoptosis of those generating large amount of ROS. Such a process can rescue the cell which otherwise would be injured by ROS.

However, if a large portion of the mitochondrial population opens their pores, this can kill the cell since pore opening inevitably entails swelling of the mitochondrial matrix and disruption of the outer mitochondrial membrane occupying a smaller area than the inner membrane. This results in a release of cytochrome *c* and a set of other apoptosis-inducing proteins normally hidden in the mitochondrial intermembrane space. The proteins in question initiate suicide of the cell (apoptosis) [200,201].

The above hypothesis predicted that the addition of ROS to a cell would have caused the thread–grain transition of the mitochondrial reticulum. Such a prediction was confirmed by the observation that the treatment of human fibroblasts with H_2_O_2_ resulted in the transformation of thread-like mitochondrial profiles to bead-like due to swelling of some parts of mitochondrial filament [193]. A subsequent study showed that direct exposure of cells to hydrogen peroxide causes either transient mitochondrial fragmentation when the oxidative challenge is transient, or significant changes in mitochondrial morphology and content when the oxidative stress is persistent [202].

The critical role of ROS in mitochondrial scission was further supported by the finding that the use of mitochondria-targeted antioxidant, SkQ1, prevented mitochondrial fragmentation and reduced the death of the cell due to the ischemia/reperfusion-induced oxidative stress [203]. However, differently from cell death due to ischemia/reoxygenation, in the apoptosis induced by UV, different signaling for apoptotic induction and mitochondrial fragmentation was found. In particular, the SkQ1, an antioxidant targeted to mitochondria, was able to prevent the reticulum of mitochondria fragmentation due to UV irradiation but was unable to prevent cell death by apoptosis [203].

The stimuli that generate ROS and induce the fragmentation of the mitochondrial network are or exogenous, for example, the treatment with pro-oxidant, chemotherapeutics, or ionizing radiation, or endogenous, for example the redox signaling produced during hypoxia.

The pathways of the stress response signaling transmit both external and internal signals between a redox-sensitive receptor and the downstream effectors of the machinery for mitochondrial fission. How these signals regulate mitochondrial fission and converge upon the apoptotic stress response is still not understood and an area of active research.

Exposing skeletal muscle myoblasts to exogenous H_2_O_2_ triggered depolarization and stimulated mitochondrial fragmentation. Furthermore, the major proteins involved in mitochondrial dynamics (Mfn1, Mfn2, OPA1, Drp1, Fis1) were surveyed, but none of them were found to be regulated transcriptionally after a 6-h treatment with 250 μM H_2_O_2_. Likewise, no significant change in Drp1 protein levels in the mitochondrial fraction was observed. These findings indicated that H_2_O_2_ does not modulate mitochondrial dynamics at the level of transcription, nor does it activate mitochondrial fission through signaling and suggested that ROS-induced mitochondrial depolarization might be responsible for mitochondrial fragmentation [204].

Conversely, another work demonstrated that oxidative stress causes activation of PKCδ leading to Drp1 phosphorylation and translocation of the Drp1/PKCδ complex to the outer mitochondrial membrane, where Drp1 binds to Fis1 [205].

In a subsequent work, it was shown that stressing myoblast with H_2_O_2_ determined a reduction in mitochondrial membrane potential followed by a 41% increase in mitochondrial reticulum fragmentation within 3 h of exposure. To define the role of dynamin-related protein 1 in H_2_O_2_-induced fragmentation, the cells were incubated with an inhibitor of dynamin-related protein 1 translocation to mitochondria, the mDivi1. Its administration attenuated H_2_O_2_-induced mitochondrial fragmentation by 27% and did not allow the changes in the levels of phosphorylated Drp1 [206].

Studying the effect of high-fluence low-power laser irradiation (HF-LPLI) revealed that HF-LPLI-induced oxidative stress and mitochondrial fragmentation were completely prevented by vitamin C pretreatment, demonstrating that the changes were mediated by oxidative stress caused by HF-LPLI. HF-LPLI also increased the mitochondrial accumulation of Drp1, which was also totally prevented by vitamin C pretreatment [207].

Moreover, in fibroblasts, exogenous H_2_O_2_ addition stimulated ubiquitination of Mfn1/Mfn2 (but not of OPA1 or hFis1) through the PINK1/Parkin pathway and promoted mitochondrial fragmentation [208]. Conversely, lowering fibroblast ROS levels by the antioxidant Trolox stimulated Mfn2-dependent mitochondrial filamentation and increased OXPHOS protein expression and enzymatic activity [209,210].

It is interesting that, whereas some studies suggest that that oxidative stress causes mitochondrial fragmentation via differential modulation of mitochondrial fission-fusion proteins [207], other studies suggest that fission may promote the mitochondrial generation of ROS [211]. It has also been suggested that changes in mitochondrial network structure provide an example of ROS-mediated ROS generation where ROS play a role in mitochondrial fission to augment ROS generation from restructured mitochondria [212].

The RNS are also involved in the regulation of mitochondrial dynamics, even though NO^•^ appears to play opposite roles in mitochondrial fission-fusion. In the process of myogenic differentiation, NO^•^ promotes the fusion of mitochondria into an elongated network by inhibiting Drp1-mediated fission [213]. Conversely, in neurodegenerative diseases, NO^•^ may enhance mitochondrial fragmentation and cell death by increasing the activity Drp1, thus promoting mitochondrial fission [214].

## 6. Removal of Damaged Mitochondria

As dysfunctional mitochondria can be dangerous for cell life, elaborate mechanisms of mitochondrial quality control have evolved to maintain a functional mitochondrial network and avoid cell damage [215]. The crucial role of these defense pathways is supported by the observation that mitochondrial dysfunction is related to a wide range of pathologies including neurodegenerative diseases [216], cancer [217,218], diabetes [219], and aging [220,221,222,223].

The cell protects itself by removing what is damaged, from mitochondria-located proteins to the same organelles by two main strategies [203,224]. In the former strategy, protein proteases remove misfolded, denatured, or oxidized proteins resident in the mitochondrial milieu [225,226], while the cytosolic ubiquitin-proteasome system recognizes and removes mistargeted and misfolded proteins before they reach the organelle and mediates the degradation of proteins embedded in the outer mitochondrial membrane [227,228].

In the latter strategy, entire parts of the same organelles are removed so that damaged mitochondria are tilted toward a fragmented phenotype, to be more disposed to segregation and removal [229,230]. On the other hand, healthy or highly active mitochondria tend to fuse among themselves in order to favor the replacement of essential components, as well as maintaining the mitochondrial genome in the network [231,232,233].

The main mechanism the cell uses to remove damaged organelles and proteins is called autophagy. This term encloses any processes regarding degradation of cytosolic components via lysosomes. The term macroautophagy indicates the process by which the engulfment and removal of cytosolic components verify. In the condition of severe mitochondrial dysfunction, a more selective process, called “mitophagy” takes place that selectively removes the damaged mitochondria.

### 6.1. Degradation of Mitochondrial Proteins

With mild damage to mitochondria, their homeostasis is maintained by the first line of defenses, acting at molecular levels, localized and operating in the organelles, constituted by several proteases and chaperones [234]. In the mitochondria, there are more than 20 proteases that are involved in diverse functions, which include processing the newly imported proteins and mitochondrial dynamics [235]. The main function of the mitochondrial proteases is to control the quality of the proteins. The proteases localized in the intermembrane space and the matrix regulate the proper ratios of the subunits of the OXPHOS complexes encoded by the nucleus and mitochondria, remove the proteins damaged or unfolded or misfolded, and regulate the protein turnover [236]. Members of the heat shock family chaperones avoid proteins synthesized in the cytosol interacting, aggregating, and entering the mitochondrion in a relatively unfolded state [236].

The cytosolic ubiquitin-proteasome system (UPS) is involved in mitochondrial quality control. Indeed, it recognizes and removes mitochondrial proteins that become a mistargeted or misfolded route to mitochondria [227]. The proteasome degrades ubiquitinated proteins on the outer mitochondrial membrane (OMM). The mitochondrial OMM proteins improperly folded are retro-translocated to the cytosol for degradation by the proteasome in a process called “outer mitochondrial membrane-associated degradation (OMMAD)” [227,228]. 

The removal of unfolded or damaged proteins localized within the mitochondria is due to a highly conserved group of proteases. Due to the activity of these proteases, the half-life of mitochondria is increased, probably for the reduced necessity to use the more energy-consuming autophagic degradation.

The quality control across the inner mitochondrial membrane is operated by two protease complexes: the membrane-bound AAA (ATPase associated with a wide variety of cellular activities) [237]. The catalytic domains of the two proteases are exposed toward the matrix (the m-AAA) or the intermembrane space (the i-AAA). Other peptidases contribute to the quality control of the inner membrane, one of them is the metallopeptidase OMA1 [238]. Moreover, an ATP serine protease, called Lon protease degrades denatured or oxidatively damaged proteins in the matrix [239,240]. Lon protease is also the only protease able to eliminate oxidatively damaged proteins [239].

The importance of the Lon proteases is demonstrated by the observation that the severe downregulation of the human Lon proteases A determines, within few days, apoptotic cell death, conversely, a less marked downregulation determines the accumulation of large and malformed mitochondria such as those normally found in aged postmitotic cells [241].

Interestingly, Lon protease also seems to play a pivotal role in the regulation of mitochondrial gene expression [242]. The depletion of Lon protease in *Drosophila* cells increases the levels of TFAM protein and mtDNA copy numbers thus increasing the transcription of mtDNA-encoded genes [242,243]. When the copy number of the mtDNA is low, Lon protease degrades TFAM to keep the TFAM/mtDNA ratio and to control the mitochondrial transcription rate. The levels in the cell of TFAM vary according to the Lon levels suggesting that Lon-mediated degradation of TFAM may regulate the levels of mtDNA levels and mtDNA transcription in physiological conditions [242,243].

### 6.2. Mitoptosis and Mitophagy

Mitophagy is the process of degradation of the harmfully damaged or no longer required mitochondria. In mammals, mitophagy contributes to eliminating functional mitochondria during starvation [244], erythroid differentiation [245], and oocyte fertilization [246]. To date, it is known that mitophagy is a universal route of elimination of dysfunctional mitochondria. The process is essential to protect the cells from the harm due to mitochondrial disordered metabolism. It is also important in attenuating apoptosis or necrosis, by preventing the inadvertent release of cytochrome c, AIF (apoptosis-inducing-factor), and other apoptotic factors.

Observations in yeast and mammalian cells, using several models of mitochondrial dysfunction, showed that fusion competence was reduced when the mitochondrial function was impaired [247,248]. This isolated dysfunctional mitochondria from the intact network, hence providing a possible way to distinguish functional from dysfunctional mitochondria on a morphological basis. It was also hypothesized that this process could target damaged mitochondria for degradation by autophagy.

Subsequent works confirmed the existence of a selection process that distinguishes ‘good’ from ‘bad’ mitochondria targeting only the latter for autophagosome engulfment, suggesting that mitochondrial dysfunction is required for the autophagic process. Indeed, autophagy of mitochondria was impaired when the fission of mitochondria was blocked [249,250].

It was also found that mitophagy was preceded by mitochondrial fragmentation and mitochondria removed by mitophagy exhibited the characteristic phenotype of fragmented organelles [250]. It was conceivable that mitochondrial fragmentation was requested because of sterical constraints, since phagosome engulfment of large or elongated mitochondria, unlike that of small fragmented organelles, would be sterically problematic.

However, the finding that mitophagy can also be induced independently from mitochondrial fission raised doubts about the fission role during mitophagy [251].

The observation that mitochondrial division produces two uneven daughter organelles, one with high membrane potential and one with decreased membrane potential and reduced OPA1 levels [249] suggest that only a fraction of daughter mitochondria produced by fission events are eliminated by autophagy. Mitochondria with decreased membrane potential and reduced OPA1 levels were less likely to be engaged in subsequent fusion events and, instead, were prone to removal by mitophagy. Remarkably, the arrest of autophagy led to the accumulation of mitochondria with low membrane potential and low OPA1.

Based on these observations, a hypothesis was proposed that integrated mitochondrial dynamics and turnover in the mitochondrial life cycle [249]. The mitochondrion cyclically shifts between a post-fusion state (Network) and a post-fission state (Solitary). Fusion is brief and triggers fission. Mitochondrial fission frequently generates solitary mitochondria that might either maintain intact membrane potential and refuse with the mitochondrial network or might be depolarized. If it depolarizes, it is unlikely to re-engage in further fusion events for the entire depolarization interval. In the case mitochondrial depolarization is transient and Δψ_m_ resumes, fusion capacity is restored. However, if Δψ_m_ depolarization is sustained, reduction in OPA1 follows and elimination by autophagy occurs. Therefore, the mitochondrial division may contribute to a quality control mechanism that facilitates the removal of damaged mitochondria from the cell.

Subsequently, the view that autophagic processes can remove damaged and dysfunctional mitochondria was directly confirmed by experiments in which selected mitochondria inside living hepatocytes were subjected to laser-induced photodamage [252]. Furthermore, such experiments confirmed that mitochondrial depolarization and inner membrane permeabilization are required for autophagy signaling [252]. This result suggests the involvement of the opening of the non-specific mitochondrial channel, called mitochondrial permeability transition (MPT) pore, and mitochondrial swelling in the mitophagy, consistently with the previous report of photodynamic induction of the MPT and involvement of the MPT in mitophagy [244].

Still, there is no consensus concerning the exact composition of MPT. Based upon biochemical and pharmacological studies, the pore was proposed to consist of the voltage-dependent anion channel (VDAC), ANT, and cyclophilin D (CypD) localized in the outer, inner mitochondrial membrane, and mitochondrial matrix, respectively [253]. However, there is now considerable evidence to indicate that the conclusions of these original studies were incorrect. Studies using transgenic VDAC1 knockout mice seem to contradict the stereotype model of MTP as it was shown that VDAC -/- were still able to undergo MPT [254]. Moreover, a mitochondrial pore-like channel, that only contained ANT and CypD and was devoid of VDAC, was reconstituted [255] and MPT can still be observed in mitoplasts that are devoid of the outer membrane [256].

Other components, such as hexokinase, benzodiazepine receptor, and creatine kinase seem to play a regulatory role rather than being structural components of the MPT.

Whatever its composition is, the pore is permeable to solutes up to 1.5 kDa so that its opening causes equilibration of H^+^ across the inner membrane, which dissipates the mitochondrial transmembrane ΔΨ_m_ and inhibits ATP production. A concomitant influx of water causes swelling of the mitochondrial matrix and disruption of the outer mitochondrial membrane occupying a smaller area than the inner membrane. Therefore, MPT pore opening inevitably entails mitochondria, which is a signal for programmed mitochondrial destruction [253], which represents a mechanism for the cell defense.

However, in some cases, when a large portion of the mitochondrial population open their pores, disruption of the outer mitochondrial membrane results in cytochrome c release and a set of other apoptosis-inducing proteins normally hidden in the mitochondrial space which initiate the cell death for apoptosis [257,258].

In the cells, mitoptosis gives rise to two different mitochondrial populations: part normal mitochondrial network and part altered or “damaged” mitochondrial entities. Of these two populations, they are the damaged mitochondria that would most likely be targeted for autophagic degradation.

Elmore and collaborators demonstrated that assimilation of the depolarized mitochondria into acidic autophagosomal/lysosomal compartments was preceded by mitochondrial depolarization by MPT [244]. Mitochondrial depolarization appears also to precede the translocation of the proteins that tag mitochondria for mitophagy such as Parkin and PTEN-induced putative kinase 1 (Pink1). Parkin is an E3 ubiquitin ligase whereas PINK1 is a mitochondrially targeted serine/threonine kinase. There are several pathways by which mitophagy is induced in mammalian cells, but even if many questions remain, the PINK1/Parkin couple is, so far, the best-characterized pathway of mitophagy of dysfunctional, depolarized mitochondria [259].

Under basal conditions, the import of PINK1 into mitochondria depends on the multiprotein complexes TOM and TIM [260]. The matrix processing peptidases (MPP) and the inner mitochondrial membrane protein, PGAM5-associated rhomboid-like protease (PARL) cleavage PINK1, which is, then, degraded by the proteasome. Mitochondrial stress or damage compromises the import of PINK1 that cannot be processed by PARL remaining on the outer mitochondrial membrane where phosphorylates the ubiquitin bound to OMM proteins. Phosphorylated ubiquitin draws Parkin from the cytosol towards the mitochondrial surface. Parkin is phosphorylated by PINK1 on Ser65 of its ubiquitin-like domain, increasing its E3 ligase activity. Activated Parkin phosphorylates OMM proteins, and PINK1 phosphorylates ubiquitin increasing Parkin recruitment and activation. Therefore, a positive feedback loop amplifying the ubiquitin phosphorylation arises and phosphorylated ubiquitin is used as a receptor by the autophagy receptor proteins.

### 6.3. MPT as a ROS Target

The signals actuating the mitoptotic program could be the ATP depletion, the block of respiration, and the depolarization of the mitochondrial membrane. However, it is possible that in all cases an overproduction of ROS is involved in the opening of the MPT pores and mitoptosis.

Since the classic studies by Haworth and Hunter, MPT is considered to be due to a proteinaceous pore opening in the inner mitochondrial membrane, an event requiring accumulation of Ca^2+^ in the mitochondrial matrix [261,262].

The first indication that MTP is due to ROS generated by mitochondria was provided by Lehningher and co-workers. They demonstrated that the Ca^2+^ efflux from mitochondria is enhanced by oxidants of mitochondrial pyridine nucleotides [263], this effect was later attributed to nonspecific inner mitochondrial membrane permeabilization [264]. The oxidants, exhausting the mitochondrial mechanism of detoxification glutathione peroxidase/glutathione, which is maintained reduced by pyridine nucleotides via glutathione reductase, determine the accumulation of H_2_O_2_ into the mitochondria [265]. Another line of evidence that MTP depends on ROS was provided by experiments showing that catalase prevents the Ca^2+^-induced MTP and that the ROS generated by a system such as a xanthine/xanthine oxidase can induce MPT [266].

Oxidative stress seems to be responsible for mitochondrial Ca^2+^ overload and promotion of MPT pore opening.

First, because of cellular oxidative stress, cytosolic Ca^2+^ increases because of its influx from extracellular space, through the plasmatic membrane, or its release from intracellular stores such as endoplasmic or sarcoplasmic reticulum. This is supported by the observation that conditions leading to increased ROS production, such as ischemia-reperfusion [267], hyperthyroidism [268], and acute exercise [269] also induce Ca^2+^ overload [270]. As mitochondria have a load of Ca^2+^ that depends on the cytosolic Ca^2+^ concentration [271], under physiological conditions, small increases in mitochondrial Ca^2^ happen that stimulate the Krebs cycle and NADH redox potential and, therefore, ATP synthesis [272]. Conversely, under oxidative stress, mitochondrial Ca^2+^ overload happens that depresses mitochondrial function [273] and primes different processes, which might lead to irreversible cell damage.

Secondly, in vitro studies suggest that, in the presence of Ca^2+^, oxidative alterations of protein thiols of mitochondrial inner membrane promote the MPT pore opening [274,275], that leads to the mitochondrial swelling. In this regard, it is interesting that the term “permeability transition” was used to designate this form of inner mitochondrial membrane protein alterations due to the reversibility of this permeabilization upon the addition of Ca^2+^ chelators and dithiol reductants soon after its onset [274,275].

Evidence exists that with the thiol groups of the ANT reduced, the MPT opening is less likely than with the ANT thiol groups oxidized, and cross-linkages formed. ANT Cys160 and Cys257 oxidation seem to be the main components involved in the MTP opening [276]. Moreover, the oxidation of the Cys56 changes the ANT conformation transforming it in a nonspecific pore similarly to what causes the Ca^2+^, which transforms ANT into a large channel [277]. As ANT is very sensitive to oxidation, mPTP is one of the principal sites of the action of the ROS in mitochondria.

### 6.4. ROS and Mitophagy

Evidence exists that ROS are involved not only in MPT but also in mitophagy which can be specifically triggered by both moderate and increased ROS levels and is conducive to cell survival, while excessive ROS can activate apoptotic death [278]. However, it has not yet been completely clarified in which phases of autophagic death ROS are involved and what their mechanism of action is.

Recent studies investigating the effects of ROS on translocation of Parkin to mitochondria have yielded conflicting results. ROS and oxidative stress have been reported to be implicated in the recruitment and localization of Parkin and DJ-1, specific proteins that are closely tied to the activation of mitophagy [279]. Indeed, ROS scavenger was able to inhibit carbonyl cyanide m-chlorophenylhydrazone (CCCP)-induced Parkin translocation in mouse embryonic fibroblasts and mouse primary cortical neurons [279]. ROS scavenger was also able to inhibit paraquat-induced Parkin translocation to mitochondria in HeLa cells [280]. However, ROS scavenger failed to attenuate CCCP-induced Parkin recruitment to mitochondria in HeLa cells [280].

More recent work has demonstrated that CCCP treatment induced both mitochondrial depolarization and generation of ROS that were needed for the mitophagy process, but mitophagy was halted following the withdrawal of CCCP treatment, even when Parkin had translocated to mitochondria. Treatment of pro-oxidant was able to propel mitophagy in the absence of further CCCP treatment, suggesting that ROS may contribute to the execution of mitophagy. However, the treatment of pro-oxidant alone was insufficient to induce translocation of Parkin to mitochondria or autophagic clearance of mitochondria. Moreover, it was also shown that the p38 signaling pathway may contribute to the progression of mitophagy induced by ROS [281]. This result agrees with those of previous authors reporting that the MAPK signaling pathway was required for mitophagy in *Saccharomyces cerevisiae* [282] and HeLa cells [283].

The possibility that ROS are also involved in autophagosome formation has been investigated and both exogenously added H_2_O_2_ [284] and O_2_^•−^ produced in the mitochondria [285] have been shown to upregulate such a formation.

Moreover, the cysteine protease HsAtg4 was identified as a direct target for oxidation by H_2_O_2_ and a cysteine residue located near the HsAtg4 catalytic site was specified as critical for this regulation [284].

A model depicting the involvement of mitochondrial H_2_O_2_ in autophagosome biogenesis has been proposed [286]. The model suggests that H_2_O_2_ released from the mitochondria create an oxidative gradient, which favors autophagosome formation at the vicinity of mitochondria and lysosomal degradation further away from the mitochondria, where reducing conditions prevail. In the oxidative environment, the protease Atg4 is inactive, enabling the formation of the autophagosome. Further away from the mitochondria, in a reducing environment, the protease cleaves and delipidates Atg8, thereby preventing the formation of new autophagosomes but enabling the recycling of this protein before degradation of the autophagosome in the lysosome.

## 7. The Closing of the Circle

In cells for which most of the ATP flux is generated through oxidative phosphorylation, the activity of oxidative phosphorylation should be maintained constant when cell energy demand does not vary. This condition can be achieved by keeping unchanged the number of the functional units of the mitochondrial oxidative phosphorylation, i.e., mitochondrial amount.

The cellular steady state of the mitochondrial amount is controlled by two processes: mitochondrial biogenesis and mitochondrial degradation, which govern the turnover of the mitochondrial compartment.

During the steady-state, the number of mitochondria present depends on the balance of mitochondrial biogenesis and degradation processes, which must be coordinated. Coordination of opposing processes of mitochondrial and clearance is primarily achieved through transcriptional and post-translational regulation of key factors. The AMPK and mTORC1 signaling pathways are two central energy sensors that can connect the regulation of mitochondrial mass to energy demands and expenditures and substrate availability. Moreover, both pathways are regulated by ROS.

### 7.1. mTORC1

Evidence is available that suggests the mammalian target of rapamycin complex 1 (mTORC1) is among the central regulators of mass and turnover of mitochondria. The normal development and growth of the cells require fine control of the cellular homeostasis and the mTORC1 signaling pathway plays a key role in its maintaining. mTORC1 integrates signals linked to the energy status and nutrient availability (i.e., amino acids, glucose, and oxygen) to control the growth of the cell [287]. Complex activation determines the phosphorylation of ribosomal protein S6 and of other factors involved in translation initiation and elongation which results in increased protein synthesis [288]. On the other hand, the inhibition of the mTORC1 signaling pathway due to limited nutrient availability or treatment with rapamycin induces the degradation of proteins and organelles through autophagic processes [287]. Inhibition of mTORC1 activity removes the repression of a complex that includes Unc-51 like autophagy activating kinase 1 (ULK1), focal adhesion kinase family interacting protein of 200-kDa (FIP200), and Atg13, resulting in autophagy. Hypoxia also hampers mTORC1 signaling through different pathways. One of these pathways requires the activation of the hypoxia-sensing protein REDD1, which localizes to the mitochondria [289]. Mitochondrial degradation occurs during mTORC1-dependent autophagy, but this is not a specific mitophagy mechanism [290]. Interestingly, increases in mTORC1-dependent autophagy are concomitant with the mitochondrial loss of energetic function. For instance, knockdown of the regulatory associated protein of mTOR (RAPTOR) and disruption of mTORC1 decrease oxygen consumption [290], and inhibition of mTORC1 with rapamycin decreases the mitochondrial ΔΨ, oxygen consumption, and cellular ATP levels and profoundly alters the mitochondrial phosphoproteome [290,291]. In parallel, mTORC1 directly controls the interaction between the transcription factor Yin-Yang 1 (YY1) and the nuclear co-factor PGC1-α, thereby regulating mitochondrial protein expression [292]. This finding highlights the key role played by this complex in mitochondrial biogenesis and oxidative metabolism [292]. Therefore, mTORC1 signaling can integrate information concerning the availability of energetic substrates to coordinate the control of both mitochondrial degradation and biogenesis. This coordination can occur at the tissue level; indeed, tissues can transiently promote either mitochondrial degradation or biogenesis, according to their energy demands. This function is exemplified in obesity and type 2 diabetes [293], which are conditions that are linked to elevated mTORC1 activation [294] but are characterized by impaired mitochondrial biogenesis in muscles [295], increased oxidative phosphorylation levels in the liver [296], and decreased phosphorylation in the heart [297].

Although many effects of ROS on mTORC1 activity are cell-type dependent, there are indications that the effect of ROS on mTORC1 is concentration-dependent. The activity of mTORC1 is induced by low levels of ROS, while mild and high levels inhibit mTORC1 activity [298]. ROS can activate mTORC1 via oxidation of cysteine groups. This is seen following treatment of cells with compounds such as phenylarsene oxide (PAO), that specifically induce disulphide bonds. Reducing agents have the opposite effect [299]. ROS and agents like PAO may either oxidize cysteine groups in the mTORC1 complex itself [88] or act at upstream levels, for example, the tuberous sclerosis complex TSC1/2 [300].

Even mechanisms by which mild and high levels of ROS inactivate mTORC1 are stimulus and cell-type dependent.

First, ROS activate stress-activated kinases, for example, JNK, that inactivate PI3K signaling via inhibitory phosphorylation of IRS1 [301]. ROS can activate AMPK via mitochondrial depolarization, which increases levels of AMP.

The inactivation of mTORC1 by ROS represents a feedback mechanism, because it can result in a reduction of the number of mitochondria via mitophagy, and thus prevent further increases in ROS formation [302]. In a similar regulatory mechanism in yeast, nitrogen starvation promotes ROS-induced mitophagy, keeping the number of mitochondria to a minimum to meet energy requirements and simultaneously prevent the production of excess ROS [303]. It should be kept in mind that the inactivation of mTORC1 by ROS is only part of an integral program by which cells prevent excessive damage. Enhanced ROS production is also counteracted by the antioxidant pathway consisting of JNK and FOXO that leads to the upregulation of manganese superoxide dismutase (MnSOD), which converts H_2_O_2_ to H_2_O and O_2_, [304]. Another important transcription factor is NRF2, which controls antioxidant genes such as g-glutamyl-cysteine ligase, involved in glutathione production. In unstressed cells, NRF2 is degraded following ubiquitination by the E3 ligase KEAP1. Oxidative stress modifies KEAP1 leading to the stabilization of NRF2 and its nuclear accumulation [305]. Interestingly, mTORC1 can stimulate NRF2 via direct phosphorylation as a feedback mechanism to protect cells against acute oxidative stress [306].

### 7.2. AMPK

Another strong candidate for involvement in the coordination of mitophagy and mitochondrial biogenesis is the AMPK signaling pathway. AMPK has been identified in the past few years to act as a central integrator of mitochondrial homeostasis by controlling various aspects of the mitochondrial life cycle, from biogenesis and dynamics to removal by mitophagy [307]. While the mTORC1 signaling pathways integrate information concerning the nutrients available in the nearby environment, the AMPK pathway senses the intracellular energy status because of its sensitivity to the intracellular ratio of AMP/ATP [308]. AMPK activates energy-producing pathways and inhibits energy-consuming processes when intracellular ATP levels are low, reflecting increased demand for energy [309]. Activation of AMPK signaling through daily muscular training or the use of pharmacological treatments with 5-aminoimidazole-4-carboxamide ribonucleotide (AICAR) [310], an analog of adenosine monophosphate able to stimulate AMPK activity, increase the cellular and tissue mitochondria content. To sustain the intracellular mitochondrial energy metabolism, AMPK can regulate both mitochondrial degradation and biogenesis. Several studies have demonstrated that AMPK signaling promotes mitochondrial biogenesis through direct phosphorylation of PGC1α [311] or via a SIRT1-dependent indirect circuit [312]. Two recent studies have shown that the activation of AMPK by glucose deprivation and AICAR treatment promotes mitophagy through the direct phosphorylation of ULK1 [313,314]. As a result, AMPK stimulates both mitophagy and mitochondrial biogenesis when energy levels are low. The consequences of the concomitant activation of mitochondrial biogenesis and mitophagy are unclear in terms of the net balance of mitochondrial contents. Based on current knowledge, several hypotheses can be put forth. For instance, AMPK-dependent mitophagy may ameliorate mitochondrial bioenergetics by preventing damage. In this case, mitophagy is only weakly activated or functions at a slow rate, and only damaged mitochondria are eliminated. This type of mechanism has been suggested for Ras homolog enriched in brain (RHEB) dependent mitophagy [315]. A second possibility is a cell- or tissue-dependent AMPK signaling [316,317]. For instance, inactivation of the AMPK pathway promotes mitophagy in oxidative skeletal muscle, resulting in increased expression of PARKIN and BCL2 Interacting Protein 3 (BNIP3), whereas its activation leads to the induction of PGC1α-dependent biogenesis [318]. The coordination of these processes may be different in other tissues (e.g., AMPK might regulate only one specific mitochondrial process). A third possibility may be that AMPK responses merge with other mitochondrial biogenesis and mitophagy signaling pathways (e.g., the mTORC1 pathway), as proposed by Kim et al. [314]. Finally, mTORC1 and AMPK control both mitochondrial biogenesis and degradation. However, details regarding the coordination of these processes are still lacking because they have mainly been investigated separately.

ROS-induced activation of AMPK is believed to be important for the beneficial effect of many medicinal drugs. For example, metformin has been reported to activate AMPK through mitochondrial-derived RNS [319]. Furthermore, ischemia/reperfusion experiments with sevoflurane protected rat hearts against ischemic injury in a manner dependent on the activation of AMPK by intracellular ROS. Application of a ROS scavenger resulted in decreased AMPK activation and diminished the cardioprotective effect of sevoflurane, thereby further substantiating the role of ROS in the activation of AMPK [320]. In mouse skeletal muscle, AMPK is activated by oxidative stress and enhances glucose transport, independent of changes in AMP or the AMP/ATP ratio [321]. Similarly, in cell culture conditions, hypoxia-induced AMPK activation is reported to be dependent on mitochondrial ROS without significant changes in the AMP/ATP ratio [322]. H_2_O_2_ has been observed to be able to strongly induce the activation of AMPK [323]. Recent studies have shown that the underlying mechanism of H_2_O_2_ activation of AMPK involves the induction of cysteine oxidation [324]. In vascular endothelial cells, it has been reported, in multiple species, that ONOO^−^ activates AMPK by a PKCζ/LKB1-dependent mechanism [325].

## 8. Conclusions

Mitochondria are essential for determining the fate of the cell because they support its energy request and regulate its dismissal directly controlling its apoptotic death. Consequently, the overall status of mitochondria is constantly monitored, allowing their number, morphology, distribution, and activity to be modulated by developmental, physiological, and environmental cues.

Sufficiently detailed molecular insights about the fate of mitochondria within the cell have been only recently acquired. The efforts of numerous researchers were needed to shed light on several proteins, including transcriptional factors and co-activators, which regulate the various phases of the mitochondrial life cycle. These findings have strengthened the view that the modifications of the mitochondrial population are part of a complex pathway ensuring the functionality of mitochondria and, therefore, of the cell. Moreover, they have allowed the development of approaches to treat diseases through triggering mitochondrial biogenesis by pharmacological manipulation.

The role of ROS and RNS in the various phases of the mitochondrial life cycle has also been appreciated in recent years. Mitochondria have long been recognized as a source of ROS in animal cells, where it is assumed that the overproduction of ROS leads to an overwhelmed antioxidant system and oxidative stress. However, several experimental studies have led to the emergence of a different perspective on the role of mitochondria and ROS they produce. This perspective views ROS as specific regulatory molecules, consistent with their role in the regulation of critical physiological processes, rather than simply as inevitable toxic by-products of aerobic metabolism. In particular, the idea is widely shared that mitochondria are an integral component of the ROS regulatory system, and that the same ROS produced in a moderate amount by mitochondria can play a crucial role in genesis, dynamics, and removal of such organelles. Despite this, the conflict concerning the mitochondrial response to antioxidant treatment shows that we still lack a comprehensive understanding of the contribution of ROS in the different phases of the mitochondrial life cycle. Moreover, other aspects concerning the role of ROS in the life cycle of mitochondria should have to be clarified. For example, because ROS are chemically heterogeneous what is the species important in the mitochondrial life cycle is not known until now. Furthermore, it is known that mitochondria possess different sites of superoxide and hydrogen peroxide production [326]. These sites may potentially act as sources of mitochondrial redox signaling that could induce different mitochondrial responses. Therefore, further studies will be necessary to clarify these points.
